# Supplementary material for: Treatment with Bacterial Biologics Promotes Healthy Aging and Traumatic Brain Injury Responses in Adult Drosophila, Modeling the Gut–Brain Axis and Inflammation Responses
Source: Cells. 2021 Apr 14;10(4):900. doi: 10.3390/cells10040900 (PMC8070821; doi:10.3390/cells10040900)
Supplement: Supplementary file 1 [file cells-10-00900-s001.pdf]

cells MDPI

Article

Treatment with Bacterial Biologics Promotes Healthy Aging and Traumatic Brain Injury Responses in Adult *Drosophila*, Modeling the Gut-Brain-Axis and Inflammation Responses

Brandon Molina [1], Jessica Mastroianni [1], Ema Suarez [1], Brijinder Soni [2], Erica Forsberg [2] and Kim Finley [1,*]

[1] Department of Biology, Shiley BioScience Center, San Diego State University, San Diego, CA 92182, USA; bmolina@sdsu.edu (B.M.); jmfmastroianni@yahoo.com (J.M.); elsuarez@sdsu.edu (E.S.)
[2] Department Chemistry and Biohemistry, San Diego State University, San Diego, CA 92182, USA; brijs-soni@gmail.com (B.S.); eforsberg@sdsu.edu (E.F.)
* Correspondence: kfinley@sdsu.edu; Tel.: +1-619-594-8980

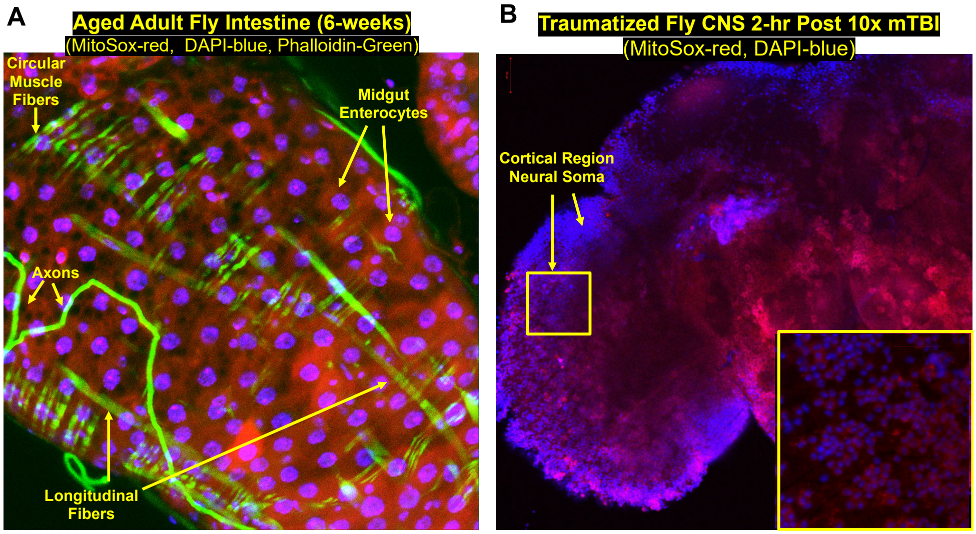

Figure S1. Elevated mitochondrial ROS levels in *Drosophila* tissues due to age or trauma (MitoSox). Adult *Drosophila* intestine (6-weeks) or CNS (2-hr Post mTBI-10x). (**A**) The GI tract from aged adult *Drosophila* (6-weeks) were dissected and stained with MitoSox Red (Superoxide Indicator, ThermoFisher Scientific, USA), Phalloidin-FITC (488nm, green, polymerized actin) and DAPI (4′, 6-diamidino-2-phenylindole, blue, DNA) for 45 mins, fixed for 30 mins (4% PFA, 1x PBS). Whole tissues were mounted and images collected using a Zeiss 710 scanning confocal microscope. Highlighted are intestinal enterocyte cells showing elevated ROS levels, along with structurally conserved axonal innervation patters as well as longitudinal and circular muscle fibers. (**B**) Young adult flies (1-week) were exposed to mTBI-10x, brains dissected 2-hours following trauma stained with MitoSox Red and DAPI for 30-mins, fixed and Z-series images collected at 20x and digitally enhanced magnifications. Highlighted are DAPI (nuclei) dense cortical regions of the fly CNS that contains neural and glial cell bodies. Inset shows elevated ROS levels in ~90% of the cell soma. Additional studies are underway to develop MitoSox staining as a quantitative estimate of total ROS levels as a result of traumatic injury or the aging process [43].

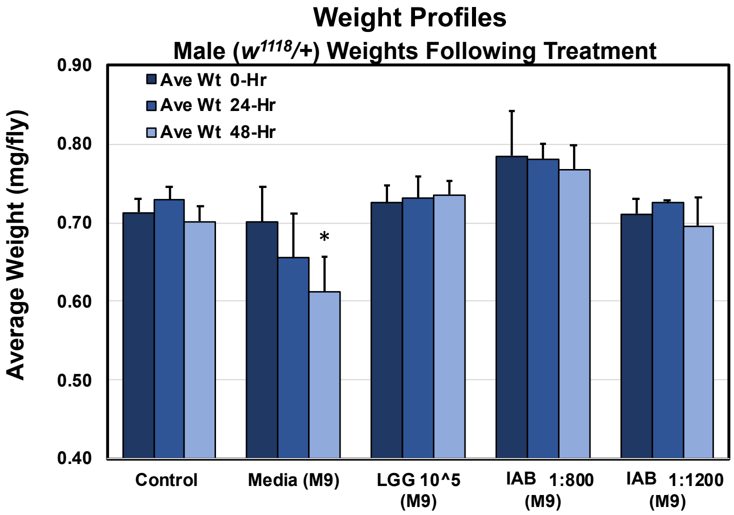

Figure S2. Adult *Drosophila* Weight profiles following treatment with bacteriologics (mg). Weights of male flies after 0, 24 and 48-h on food containing M9 media, LGG (10^5 CFUs) or the IAB (1:800, 1:1,200, Adult *Drosophila* intestinal (six weeks) and neural (2 h post-10× mTBI) tissues and stained with Mitosox to highlight mitochondrial OS profiles; * *p*-value < 0.5) [11].

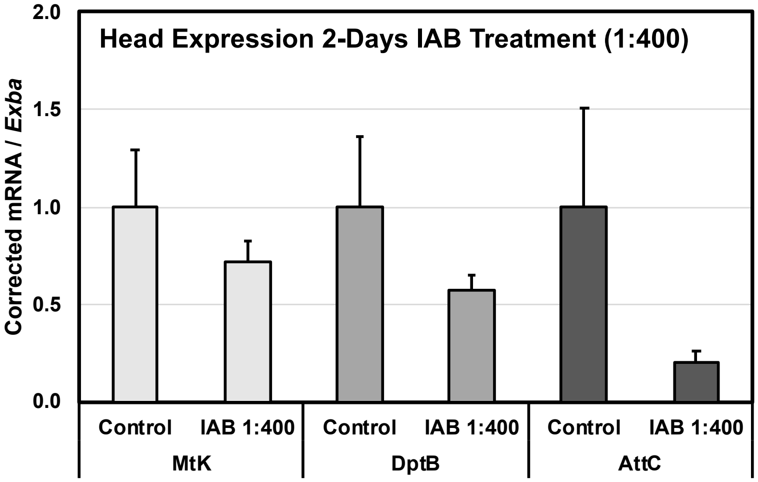

Head AMP Expression Levels 2-Days IAB Treatment				
Gene	Treatment	Ave	SEM (9)	P value
MtK	Control	1.000	0.296	
	IAB 1:400	0.723	0.105	0.3913
DptB	Control	1.000	0.364	
	IAB 1:400	0.574	0.077	0.268
AttC	Control	1.000	0.508	
	IAB 1:400	0.208	0.053	0.1407

Figure S3. Expression profiles of NFKβ pathway components following IAB treatment. Triplicate cohorts of young flies (30) were placed on control or IAB (1 to 400 dilution) treated media for 2-days. Each cohort was collected, flash frozen, and RNA isolated from heads. Individual cDNA libraries were assessed in triplicate (*n* = 9). *Exba* (loading control), *Mtk*, *DptB* and *AttC* primer sets (antimicrobial peptides, AMPs) for quantitative RT-PCR analysis to detect gene expression changes in the fly CNS. While not reaching statistical significance, a downward trend was seen in each AMP message levels, along with a reduction in expression variance between cDNA library pools [9].

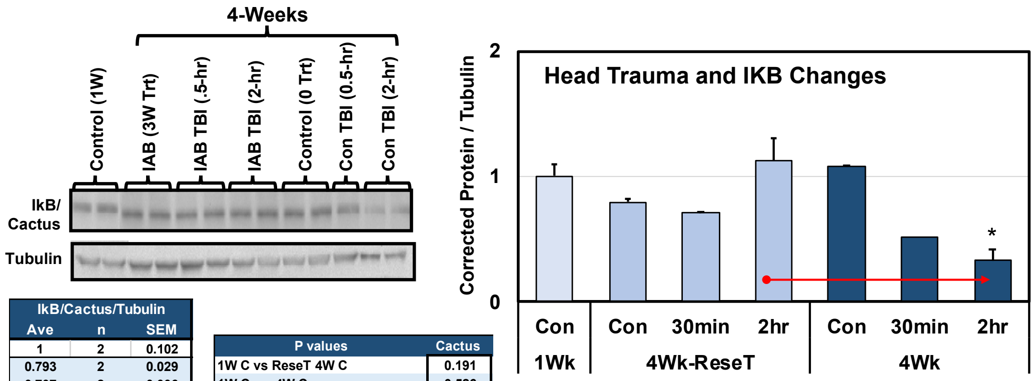

IkB/Cactus/Tubulin		
Ave	n	SEM
1	2	0.102
0.793	2	0.029
0.707	2	0.006
1.126	2	0.182
1.078	2	0.003
0.511	1	
0.327	2	0.088

P values	Cactus
1W C vs ReseT 4W C	0.191
1W C vs 4W C	0.526
4WReseT vs 4W ReseT 30m	0.101
4WReseT vs 4W ReseT 2h	0.214
4WReseT 30m vs 4W ReseT 2h	0.149
4W ReseT 2h vs 4W C 2h	0.059

Figure S4. Western analysis of NFKβ pathway components with age, IAB treatment and trauma. Total neural (heads) protein extracts prepared from 1-week and 4-week-old control and IAB treated (1:800, 3-wks) fly cohorts. Flies were collected and flash frozen 0, 30-mins or 2-hrs following mTBI-10x exposure, heads collected and total protein extracts used for Western blots that were probed with *Drosophila* Cactus (3H12, Developmental Studies Hybridoma Bank, DSHB, https://dshb.biology.uiowa.edu) and Tubulin (E7, DSHB) antibodies (*p* value *<0.5). Blots were developed using Thermo Scientific West Dura Substrate (ThermoFisher Scientific, www.thermofisher.com), ChemiDoc digital Imaging System and Quantity One software (Bio-Rad). The intensities of each protein band were quantified using ImageJ software (imagej.nih.gov/ij/). *Drosophila* trauma studies showed dynamic alterations to immune pathway targets (AMPs) in adult neural tissues. NF-Kβ pathway activation typically requires targeted proteolytic turnover of the pathway inhibitor, I-Kβ (Cactus). Studies using young flies exposed to mTBI-10x showed a rapid reduction (30-min) and recovery (2-hour) of neural IKβ (Cactus) protein levels. Western blots showed aged controls flies have impaired 2-hr rebound in I-Kβ levels while IAB treated (1:800) fly cohorts (4-weeks) had more youthful rebound of Cactus levels. This indicates IAB treatment promotes normal NFKβ pathway responses at an age where the regulation of multiple proteolytic and regulatory pathways shows significant dysregulation in adult *Drosophila* [42].

Table S1A. Summary of NGR and longevity profiles for IAB-treated flies.

1-Week Males		Ave	STD	n	SEM	% Change	
Dilutions	1Wk	4.9	0.34	597	0.0139	From 1Wk	
Control	2Wk	4.29	0.366	96	0.0374	-12.5	
	3Wk	3.16	0.392	89	0.0416	-35.6	
	4Wk	3.14	0.402	79	0.0452	-35.9	From Weekly Control
	5Wk	2.86	0.302	60	0.039	-41.6	
1 to 50	2Wk	4.25	0.326	97	0.0331	-13.2	-0.85
	3Wk	3.47	0.381	92	0.0398	-29.2	9.85
	4Wk	3.16	0.46	72	0.0542	-35.5	0.53
	5Wk	2.69	0.239	53	0.0328	-45.1	-5.9
1 to 100	2Wk	4.48	0.432	93	0.0448	-8.5	4.59
	3Wk	4.04	0.58	85	0.0629	-17.5	28.04
	4Wk	3.93	0.585	80	0.0654	-19.7	25.18
	5Wk	3.04	0.471	59	0.0613	-38	6.3
1 to 200	2Wk	4.53	0.326	98	0.0329	-7.5	5.77
	3Wk	4.03	0.458	92	0.0478	-17.8	27.56
	4Wk	3.96	0.404	81	0.0449	-19.1	26.21
	5Wk	3.13	0.301	64	0.0377	-36.2	9.38
1 to 400	2Wk	4.52	0.245	100	0.0245	-7.7	5.47
	3Wk	4.32	0.36	93	0.0373	-11.8	36.83
	4Wk	3.73	0.414	85	0.0449	-23.9	18.66
	5Wk	3.05	0.306	62	0.0388	-37.7	6.83
1 to 800	2Wk	4.55	0.396	99	0.0398	-7.1	6.19
	3Wk	4.16	0.402	97	0.0409	-15.1	31.83
	4Wk	3.85	0.341	92	0.0356	-21.5	22.47
	5Wk	3.11	0.297	79	0.0335	-36.6	8.68

Table S1B. IAB Treatment and Adult *Drosophila* Longevity Profiles.

Lifespan Profiles Trial 1							Lifespan Profiles Trial 2		
Dilution	Control	1to50	1to100	1to200	1to400	1to800	Control 2	1to1200	1to1600
Ave (days)	41.39	37.64	40.06	41.49	39.52	45.34	42.64	52.45	48.88
STD	8.69	9.40	8.39	10.93	8.63	7.45	11.95	12.94	16.11
N	80	91	80	85	89	83	176	75	85
SEM	0.972	0.985	0.938	1.186	0.915	0.818	0.901	1.494	1.748
% Difference		-9.06%	-3.20%	0.26%	-4.50%	9.54%		23.02%	14.65%
P value		0.00770	0.32793	0.94502	0.16273	0.00215		4.439E-05	1.098E-02

See manuscript Figure 1 for additional details.

Table S2. Summary of NGR Profiles of IAB-treated flies following mTBI-10×.

A. Geotaxis profiles 2-day IAB pre-treated exposed to mTBI 10×.

2-Days IAB Pre-Treat			Avg	STD	n	SEM	% Change		
1 Week			4.75	0.365	534	0.0158	From 1Wk	From 2Wk	From TBI
2 Weeks	Control	non-TBI	4.15	0.357	145	0.0297	-12.7		
1Wk Post-TBI		TBI	3.57	0.388	82	0.0428	-24.8	-13.9	
		1 to 400	3.97	0.311	92	0.0324	-16.4	-4.3	11.1***
		1 to 800	4.29	0.44	79	0.0495	-9.8	3.3	20***
*** P value <0.001		1 to 1200	4.19	0.398	92	0.0415	-11.9	0.9	17.2***

B. Geotaxis profiles 5-day IAB pre-treated exposed to mTBI 10×

5-Days IAB Pre-Treat			Avg	STD	n	SEM	% Change		
1 Week			4.69	0.421	562	0.0177	From 1Wk	From 2Wk	From TBI
2Weeks	Control	non-TBI	4.49	0.302	116	0.028	-4.2		
1Wk Post-TBI		TBI	3.76	0.388	77	0.0443	-19.8	-16.2	
		1 to 400	3.55	0.439	81	0.0487	-24.3	-20.9	-5.6
		1 to 800	3.98	0.276	84	0.0301	-15.2	-11.4	5.7 ***
*** P value <0.001		1 to 1200	4.06	0.317	68	0.0384	-13.4	-9.6	8 ***

C. Geotaxis profiles exposed to mTBI-10× post-treated.

Post IAB Treat			Avg	STD	n	SEM	% Change		
1 Week			4.75	0.365	534	0.0158	From 1Wk	From 2Wk	From TBI
2Wks	Control	non-TBI	3.57	0.421	172	0.0321	-24.8		
1Wk Post-TBI		TBI	3.16	0.174	79	0.0196	-33.5	-11.7	
		1 to 100	3.43	0.392	85	0.0426	-27.8	-4	8.7 ***
		1 to 400	3.84	0.522	82	0.0577	-19.1	7.5	21.7 ***
*** P value <0.001		1 to 800	3.76	0.279	74	0.0325	-20.8	5.3	19.2 ***

See manuscript Figure 3 for additional details.

Table S3. Summary of longevity profiles of IAB-treated flies following mTBI-10×.

A. Lifespan profiles of 2-day IAB pre-treated male flies exposed to mTBI-10× at 1-week

2-days Pre-Treat	Average	STD	n	SEM	% Change		*P-values*	
Control	46.6	12.73	119	1.17	Control	TBI Cont	Control	TBI Cont
mTBI 10x	33.4	9.79	80	1.04	-28.32		<0.0001	
1 to 400	37.0	11.70	84	1.27	-20.64	10.43	<0.0001	0.051
1 to 800	31.8	12.37	70	1.48	-31.72	-5.00	<0.0001	0.3571
1 to 1200	31.3	9.78	82	1.08	-32.82	-6.52	<0.0001	0.1577

B. Lifespan profiles of 5-day IAB pre-treated male flies exposed to mTBI-10× at 1-week

5-days Pre-Treat	Average	STD	n	SEM	% Change		*P-values*	
Control	42.3	13.36	99	1.343	Control	TBI Con	Control	TBI Con
Control mTBI	25.4	3.60	87	0.386	-39.85		<0.0001	
1 to 400	26.9	4.17	84	0.455	-36.44	5.68	<0.0001	0.0163
1 to 800	27.8	4.88	79	0.549	-34.30	9.23	<0.0001	0.0005
1 to 1200	26.8	4.09	66	0.503	-36.63	5.36	<0.0001	0.0303

C. Lifespan profiles of male flies exposed to mTBI-10× at 1-week and post-treated

Post-Treatment	Average	STD	n	SEM	% Change		*P-values*	
Control	41.5	12.03	151	0.979	Control	TBI Cont	Control	TBI Cont
Control mTBI	28.1	5.07	83	0.557	-32.43		<0.0001	
1 to 100	26.6	3.29	87	0.353	-35.89	-5.13	<0.0001	0.0287
1 to 400	26.7	3.73	87	0.400	-35.64	-4.76	<0.0001	0.0512
1 to 800	27.8	5.67	74	0.660	-33.09	-0.98	<0.0001	0.7489

See manuscript Figure 4 for additional details.

Table S4. Summary of NGR and longevity profiles for IAB- and LGG-treated flies.

A. Negative Geotaxis Responses with IAB or LGG Treatments

Treatment at 1-Week	Climbing Index	N	SEM	*P Values*	% Change
**Pre-Treatment (1W)**	4.247	297	0.027		
**Controls M9 (2W)**	4.08	69	0.052	0.0072	-3.9
**LGG 10^5 (2W)**	4.461	73	0.053	>0.0001*	9.3
**IAB 1:800 (2W)**	4.534	73	0.037	>0.0001*	11.1
**IAB 1:1200 (2W)**	4.525	76	0.028	>0.0001*	10.9
**Treatment at 3 Weeks**	**Climbing Index**	**N**	**SEM**	***P Values***	**% Change**
**Pre-Treatment (3W)**	3.547	269	0.023		
**Controls M9 (4W)**	3.401	66	0.031	0.0035*	-4.1
**LGG 10^5 (4W)**	3.621	47	0.066	0.0013*	6.5
**IAB 1:800 (4W)**	3.936	75	0.038	>0.0001*	15.7
**IAB 1:1200 (4W)**	3.915	63	0.068	>0.0001*	15.1

* p values between age-matched M9 controls and treated fly cohorts

B. Adult *Drosophila* Longevity Profiles with IAB or LGG Treatments at 1 and 3-weeks of age

	Treatment Starting at 1-Week			Treatment Starting at 3-Weeks			
	Control (M9)	ReseT (1:800)	LGG (10^5)	Control (M9)	LGG 10^5	ReseT 1:800	ReseT 1:1200
Ave (Days)	29.19	31.44	29.14	40.523	46.154	42.052	43.724
STD	10.65	11.534	8.33	8.018	7.002	7.36	7.608
N	75	76	74	65	39	74	76
SEM	1.23	1.323	0.968	0.994	1.121	0.856	0.873
P values		0.2151	0.9746		0.0004	0.2433	0.0164
% Change		7.70%	0.17%		13.89%	3.78%	7.33%

See manuscript Figure 5 for additional details.

Table S5. Summary of NGR profiles IAB- or LGG- treated flies following mTBI-10×.

	2-Weeks				
**Treatment**	**1W Treated**	**n**	**SEM**	**P values**	**% Change**
**Control (M9)**	**4.47**	**99**	**0.039**		
**LGG 10^5**	**4.21**	**94**	**0.048**	0.0001	**-5.8**
**IAB 1:800**	**4.41**	**181**	**0.034**	**0.3145**	**4.8**
	**3-Weeks**				
	**1W Post mTBI (10x)**	**n**	**SEM**	**P Values**	**% Change**
**Control (M9)**	**3.1**	**62**	**0.053**		
**LGG 10^5**	**3.42**	**73**	**0.056**	>0.0001	**10.3**
**IAB 1:800**	**3.89**	**58**	**0.054**	>0.0001	**13.7**
	**4-Weeks**				
	**2W Post mTBI (10x)**	**n**	**SEM**	**P Values**	**% Change**
**Control (M9)**	**1.56**	**31**	**0.143**		
**LGG 10^5**	**2.97**	**43**	**0.078**	>0.0001	**90.4**
**IAB 1:800**	**3.14**	**42**	**0.086**	>0.0001	**101.3**

See manuscript Figure 6 for additional details.
